# K_V_7 but not dual small and intermediate K_Ca_ channel openers inhibit the activation of colonic afferents by noxious stimuli

**DOI:** 10.1152/ajpgi.00141.2023

**Published:** 2023-09-05

**Authors:** Charity N. Bhebhe, James P. Higham, Rohit A. Gupta, Tim Raine, David C. Bulmer

**Affiliations:** ^1^Department of Pharmacology, University of Cambridge, Cambridge, United Kingdom; ^2^Department of Gastroenterology, Addenbrookes Hospital, Cambridge University Teaching Hospitals, Cambridge, United Kingdom

**Keywords:** colonic nociception, IKCa channels, K_V_7 channels, SKCa channels, visceral pain

## Abstract

In numerous subtypes of central and peripheral neurons, small and intermediate conductance Ca^2+^-activated K^+^ (SK and IK, respectively) channels are important regulators of neuronal excitability. Transcripts encoding SK channel subunits, as well as the closely related IK subunit, are coexpressed in the soma of colonic afferent neurons with receptors for the algogenic mediators ATP and bradykinin, P2X3 and B_2_, highlighting the potential utility of these channels as drug targets for the treatment of abdominal pain in gastrointestinal diseases such as irritable bowel syndrome. Despite this, pretreatment with the dual SK/IK channel opener SKA-31 had no effect on the colonic afferent response to ATP, bradykinin, or noxious ramp distention of the colon. Inhibition of SK or IK channels with apamin or TRAM-34, respectively, yielded no change in spontaneous baseline afferent activity, indicating these channels are not tonically active. In contrast to its lack of effect in electrophysiological experiments, comparable concentrations of SKA-31 abolished ongoing peristaltic activity in the colon ex vivo. Treatment with the K_V_7 channel opener retigabine blunted the colonic afferent response to all applied stimuli. Our data therefore highlight the potential utility of K_V_7, but not SK/IK, channel openers as analgesic agents for the treatment of abdominal pain.

**NEW & NOTEWORTHY** Despite marked coexpression of small (*Kcnn1*, *Kcnn2*) and intermediate (*Kcnn4*) conductance calcium-activated potassium channel transcripts with P2X3 (*P2rx3*) or bradykinin B_2_ (*Bdkrb2*) receptors in colonic sensory neurons, pharmacological activation of these channels had no effect on the colonic afferent response to ATP, bradykinin or luminal distension of the colon. This is in contrast to the robust inhibitory effect of the K_V_7 channel opener, retigabine.

## Graphical abstract

**Figure:**
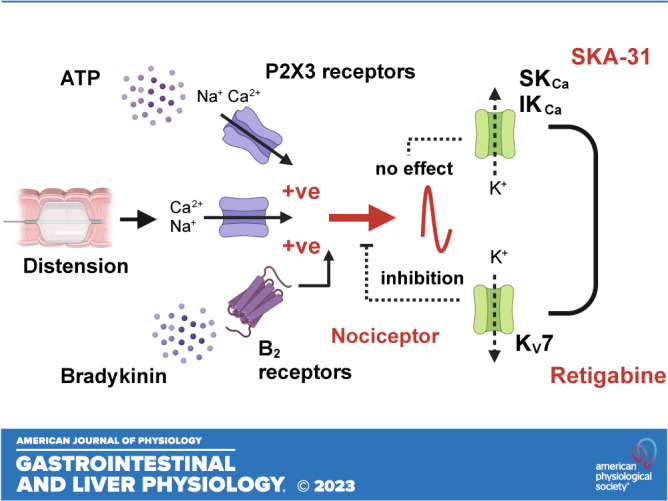

## INTRODUCTION

Ca^2+^-activated K^+^ (K_Ca_) channels couple intracellular Ca^2+^ signals to K^+^ efflux, thereby regulating membrane excitability in nerve and muscle, and cell volume and K^+^ homeostasis in nonexcitable cells. The α subunits of these channels are split into three subclasses based on single channel conductance: small conductance Ca^2+^-activated K^+^ channels (SK1-3, 4-14 pS) encoded by *Kcnn1-3* (1), intermediate conductance Ca^2+^-activated K^+^ channels (IK, 30-40 pS) encoded by *Kcnn4* (2) and large conductance Ca^2+^-activated K^+^ channels (BK, 200-300 pS) encoded by *Kcnma1* (3). Members of the K_Ca_ channel family also differ in their Ca^2+^ sensitivity, coupling to different Ca^2+^ sources, voltage dependence (only BK is voltage-dependent), and pharmacological properties. SK channel α subunits have distinct but overlapping tissue expression, with functional channels formed of α subunit tetramers that can assemble as both homomers and heteromers, providing greater diversity within this family of K^+^ channels (4, 5).

SK channel subunits are expressed in peripheral sensory neurons (6, 7), and there is now evidence for these channels playing a role in the processing of both innocuous and noxious stimuli. Immunofluorescent staining has shown coexpression of SK1 and SK2 channels with peptidergic and nonpeptidergic markers in small-diameter sensory neurons in rat dorsal root ganglia (DRG), whereas SK3 is more broadly expressed (8). Sensory input to the spinal cord during innocuous and noxious mechanical stimulation was elevated by inhibition of SK channels, whereas SK channel activation attenuated sensory input (9). IK channels are also expressed in small-diameter sensory neurons (8). What’s more, genetic ablation or pharmacological blockade of IK channels potentiated nocifensive behavior following formalin or capsaicin administration, but had no effect on inflammatory or neuropathic pain (10).

Sensory signaling from the distal colon and rectum is mediated by the lumbar splanchnic and pelvic nerves and is of vital importance for defecation and the sensation of bloating, urgency, and pain (11). A greater understanding of the ion channels that regulate colonic afferent activity will provide insights into the mechanisms underpinning visceral sensation and may highlight potential targets for future therapeutics. Whether SK/IK channels regulate colonic afferent function is currently unknown.

In this study, we have used pharmacological tools to compare the effect of SK/IK and K_V_7 channel activation on the colonic afferent response to algogenic stimuli and noxious distention of the colon. Our data demonstrate that pretreatment with the SK/IK channel opener SKA-31 at concentrations that abolished peristaltic activity in the colon had no effect on the colonic afferent response to ATP, bradykinin (BK), or noxious distention of the colon. In alignment with a previous report, subsequent activation of K_V_7 channels by retigabine (RTG) robustly inhibited the colonic afferent response to each stimulus (12). These data highlight the potential utility of K_V_7, but not SK/IK, channel openers for the treatment of abdominal pain.

## METHODS AND MATERIALS

### Animals

Experiments with ATP were performed using tissue from female CD1 mice, whereas all other experiments were performed with male C57Bl/6 mice (all 10–14 wk of age) euthanized by exposure to rising concentrations of CO_2_ followed by cervical dislocation in accordance with schedule 1 of the Animals (Scientific Procedures) Act 1986 Amendment Regulations 2012 and local ethical review by the University of Cambridge Animal Welfare and Ethical Review Body (AWERB). Mice were housed in cages of up to five littermates under a 12-h light/dark cycle with enrichment (e.g., igloos and tunnels) and ad libitum access to food and water.

### In Silico Analysis of SK/IK Transcript Expression in Colon Projecting Sensory Afferents

To establish the expression of transcripts encoding SK and IK channel subunits, as well as receptors for ATP and bradykinin, we used a previously published, publicly available RNAseq dataset comprising back-labeled colonic sensory neurons (13). Transcript expression is given as the base ten logarithm of transcripts per million. The proportion of colonic afferents coexpressing given transcripts was ascertained manually using conditional counting functions in Microsoft Excel.

### Colonic Afferent Fiber Studies

After euthanasia, the colorectum (from splenic flexure to anus) with the associated lumbar splanchnic nerve was isolated, removed, and luminal content gently flushed. The tissue was transferred to a recording bath where it was cannulated to allow luminal perfusion against an end pressure of 2–5 mmHg (200 µL/min) and serosally superfused (7 mL/min; 32°C–34°C) with Krebs buffer (in mM: 124 NaCl, 4.8 KCl, 1.3 NaH_2_PO_4_, 25 NaHCO_3_, 1.2 MgSO_4_, 11.1 d-glucose, and 2.5 CaCl_2_) supplemented with atropine (10 µM) and nifedipine (10 µM) to block smooth muscle contractility. Ongoing nerve discharge was recorded from isolated lumbar splanchnic nerve bundles (rostral to the inferior mesenteric ganglia) using borosilicate glass suction electrodes. Signals were amplified (gain, 5,000), band-pass filtered (100–1,500 Hz, Neurolog, Digitimer, Ltd., UK), and digitally filtered for 50 Hz noise (Humbug, Quest Scientific, Canada) before being digitized (20 kHz, Micro1401, Cambridge Electronic Design, UK) and recorded using Spike2 software and displayed in a chart recorder format (Cambridge Electronic Design, UK). Nerve activity was quantified by counting the number of action potentials (spikes) passing a threshold set at a magnitude of twice the background noise and displayed as a rate histogram using the data analysis function in spike2. Luminal pressure was recorded using a pressure transducer (Neurolog NL108) filled with Krebs buffer, signals were digitized at 100 Hz and displayed alongside nerve activity using spike 2.

Changes in the colonic afferent response to ATP, bradykinin, and ramp distension (0–80 mmHg; performed by occluding the luminal outflow) were determined in separate tissues in the presence of pretreatment with the SK/IK channel opener SKA-31 (100 µM, Tocris, Cat. No. 3670) and the K_V_7 channel opener retigabine (100 µM, Tocris, Cat. No. 6233), or respective vehicle control (DMSO 0.1%). For studies with ATP, colonic afferent responses were measured to three separate applications of ATP (20 mL, 3 mM, Sigma, Cat. No. A26209) given 60 min apart. The second application of ATP was given in the presence of SKA-31 or vehicle and was preceded by a 50-mL pretreatment with SKA-31 or vehicle. The third application of ATP was given in the presence of retigabine or vehicle and was preceded by a 50-mL pretreatment with retigabine or vehicle. For studies with BK, colonic afferent responses were measured to four separate applications of bradykinin (20 mL, 1 µM, Sigma, Cat. No. 05-23-0500) given 30 min apart. The third application of bradykinin was given in the presence of SKA-31 or vehicle and was preceded by a 50-mL pretreatment with SKA-31 or vehicle. The fourth application of ATP was given in the presence of retigabine or vehicle and was preceded by a 50-mL pretreatment with retigabine or vehicle. For ramp distensions, the colonic afferent response to distension (0–80 mmHg, evoked by 200 µL/min luminal perfusion with Krebs buffer) were measured in response to seven separate distensions given 15 min apart. The fourth ramp distension was performed in the presence of SKA-31 or vehicle and was preceded by a 7-min pretreatment with SKA-31 or vehicle. The sixth ramp distension was performed in the presence of retigabine or vehicle and was preceded by a 7-min pretreatment with retigabine or vehicle. In addition, the effect of vehicle (50 mL, 0.1% DMSO), TRAM-34 (50 mL, 10 µM, Sigma, Cat. No. 2946), and apamin (50 mL, 1 µM, Sigma, Cat. No. 1652) on ongoing nerve discharge was examined in separate experiments.

### Data Analysis

For tissues stimulated with either ATP or bradykinin, colonic afferent response curves were generated by subtracting baseline nerve activity, calculated as the mean nerve discharged over the 3-min period before administration of ATP or BK, from the ongoing nerve discharge measured in 1-min intervals from 6 min before administration of ATP or BK to 24 min afterward (30 min in total). Following this, the effect of drug pretreatment was examined by comparing mean changes in afferent activity for time-matched preparations pretreated with SKA-31 versus vehicle and retigabine versus vehicle using unpaired Student’s *t* test for comparison of peak changes in afferent activity and two-way ANOVA for response profiles over time. The area under afferent response profiles was calculated using the trapezoidal method and was used to examine the desensitization of the afferent response to repeated applications of ATP or BK.

For tissue stimulated by ramp distension, colonic afferent response curves were generated by subtracting baseline nerve activity, calculated as the mean nerve activity over the 3-min period before luminal distension, from the ongoing nerve discharge measured in 5-mmHg intervals to 80 mmHg. From these curves, the effect of respective drug pretreatment (SKA-31 or retigabine) was statistically compared with vehicle-treated preparations using a two-way ANOVA, and the peak response to colorectal distension at 80 mmHg following drug pretreatment (normalized to the peak response obtained from the distention before treatment) statistically compared with respective time-matched vehicle pretreatment using an unpaired Student’s *t* test.

Changes in ongoing nerve discharge were also examined following pretreatment with the IK channel blocker TRAM-34, the SK channel blocker apamin, and compared with respective changes in ongoing nerve discharge following pretreatment with vehicle. The effect of SKA-31 or retigabine on ongoing nerve discharge (before treatment with bradykinin or ATP) was also investigated. Changes in ongoing nerve discharge were generated by subtracting baseline nerve activity, calculated as the mean nerve activity over the 3-min period before administration of respective treatments, from the ongoing nerve discharge measured in 1-min intervals from 3 min before administration to 6 min afterward. Changes were statistically compared between respective vehicle and treatment groups using a two-way ANOVA with Holm–Sidak post hoc tests between time points.

### Colonic Motility

After euthanasia, the descending colon and rectum were removed and cannulated in a tissue bath superfused with Krebs buffer (7 mL/min; 32°C–34°C) and luminally perfused with Krebs buffer in an open system at a rate of 200 µL/min. Luminal pressure was monitored using a Neurolog NL108 pressure transducer, with the signals digitized (100 Hz, Micro1401, Cambridge Electronic Design, UK) and recorded using Spike2 (Cambridge Electronic Design, UK). Luminal pressure was raised to 5–10 mmHg by elevation of the outflow to trigger colonic migrating motor complexes (CMMCs). Tissue was stabilized for at least 30 min before drug application (SKA-31, 70 mL, 100 µM). The amplitude of CMMCs in each experiment was determined by averaging the peak change in pressure for all individual CMMCs before and after drug application. The average CMMC frequency (per minute) before and after drug application was also determined. Changes in the amplitude and frequency of CMMCs were statistically compared before and after drug treatment using a paired Student’s *t* test.

### Data Analysis and Statistics

All graphing and analysis were carried out in Prism 9 (GraphPad, Inc.). Data were scrutinized to ensure they met the assumptions of parametric analyses and, where appropriate, nonparametric rank-based analyses were used. Sample sizes were not predetermined before data acquisition, but intergroup comparisons were decided before experiments began. *P* value cutoffs are displayed in figures as *P < 0.05, **P < 0.01, ***P < 0.001, and ****P < 0.0001.

## RESULTS

### Expression of SK and IK Channels in Colonic Afferent Neurons

**Figure 1.**
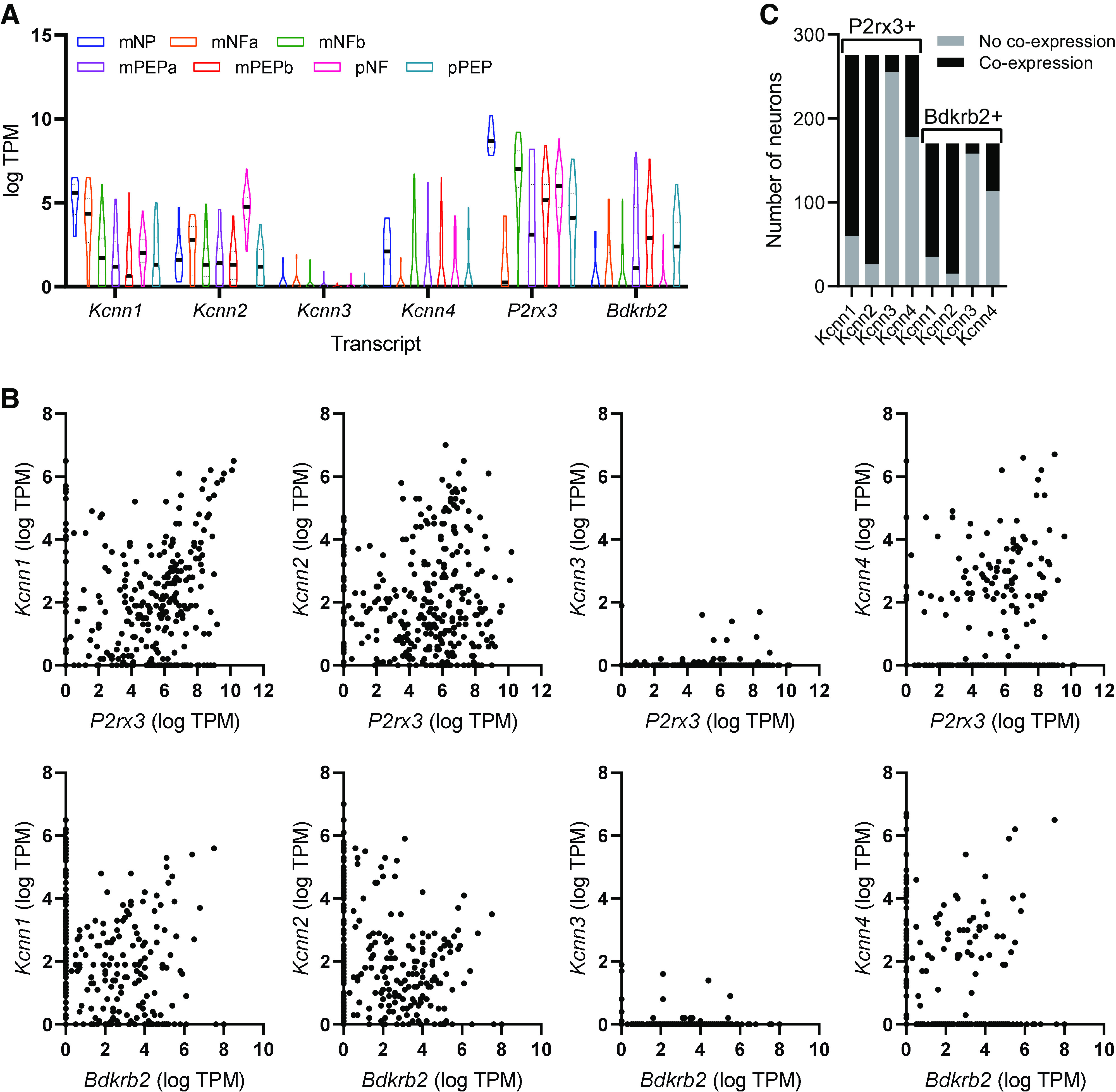
Expression of transcripts encoding small conductance Ca^2+^-activated K^+^ (SK) and intermediate conductance Ca^2+^-activated K^+^ (IK) channel subunits in colonic sensory neurons. *A*: expression (expressed as transcripts per million, TPM) of transcripts encoding SK channel subunits and receptors for ATP (*P2rx3*) and bradykinin (*Bdkrb2*) across colonic sensory neuron subtypes. *B*: neuronal subtypes are denoted: NP, nonpeptidergic; NFa/b, neurofilament expressing; PEP, peptidergic; m, mixed lumbar splanchnic and pelvic afferents; p, pelvic afferents. Scatter plots showing the coexpression of *P2rx3* (*top row*) and *Bdkrb2* (*bottom row*) with *Kcnn1–4*. Each point represents a single colonic afferent neuron. *C*: grouped data showing the number of neurons expressing either *P2rx3* or *Bdkrb2*, which coexpress *Kcnn1–4*. Data redrawn from Hockley et al. (13).

To ascertain the expression of transcripts encoding SK channel genes, we used previously published single-cell RNAseq data from back-labeled murine colonic afferent neurons (13). The expression of *Kcnn1–4* was examined across all colonic afferent subtypes (Fig. 1*A*). While *Kcnn1–2* were broadly expressed in all colonic afferent subtypes, *Kcnn3* was poorly expressed (Fig. 1*A*). *Kcnn4* was also expressed in colonic afferent neurons, though not to the same extent as *Kcnn1* and *Kcnn2* and was primarily expressed in nonpeptidergic afferents. We also examined the expression of transcripts encoding receptors for ATP (P2X_3_, *P2rx3*) and bradykinin (B_2_ receptor, *Bdkrb2*) as these algogenic stimuli will be used later in this study. *P2rx3* was widely expressed across colonic afferent subtypes, whereas *Bdkrb2* was more selectively expressed in peptidergic afferents (Fig. 1*A*).

There was notable coexpression of *Kcnn1–2*, but less so of *Kcnn3–4*, with *P2rx3* and *Bdkrb2* (Fig. 1*B*). Two hundred seventy-six colonic afferent neurons expressed *P2rx3*, of which 216 (78.3%) and 250 (90.6%) coexpressed *Kcnn1* and *Kcnn2*, respectively (Fig. 1*C*). *Bdkrb2* was expressed in 170 colonic afferent neurons, of which 135 (79.4%) and 155 (91.2%) coexpressed *Kcnn1* and *Kcnn2*, respectively (Fig. 1*C*). *Kcnn3* was coexpressed in 7.6% and 7.1% of *P2rx3*- and *Bdkrb2*-expressing colonic afferent neurons, respectively (Fig. 1*C*). *Kcnn4* was coexpressed in 35.5% and 33.5% of *P2rx3*- and *Bdkrb2*-expressing colonic sensory neurons, respectively (Fig. 1*C*).

Although P2X3 is the dominant ionotropic ATP receptor expressed by colonic sensory neurons, metabotropic ATP receptors (e.g., P2Y_1_) are also expressed by these afferents (13, 14). Of 98 neurons expressing *P2ry1* (encoding P2Y_1_), 81 (82.6%), 91 (92.3%), 7 (7.1%), and 35 (35.7%) coexpressed *Kcnn1*, *2*, *3*, and *4*, respectively.

### SK/IK Channels Do Not Regulate the Afferent Response to ATP

**Figure 2.**
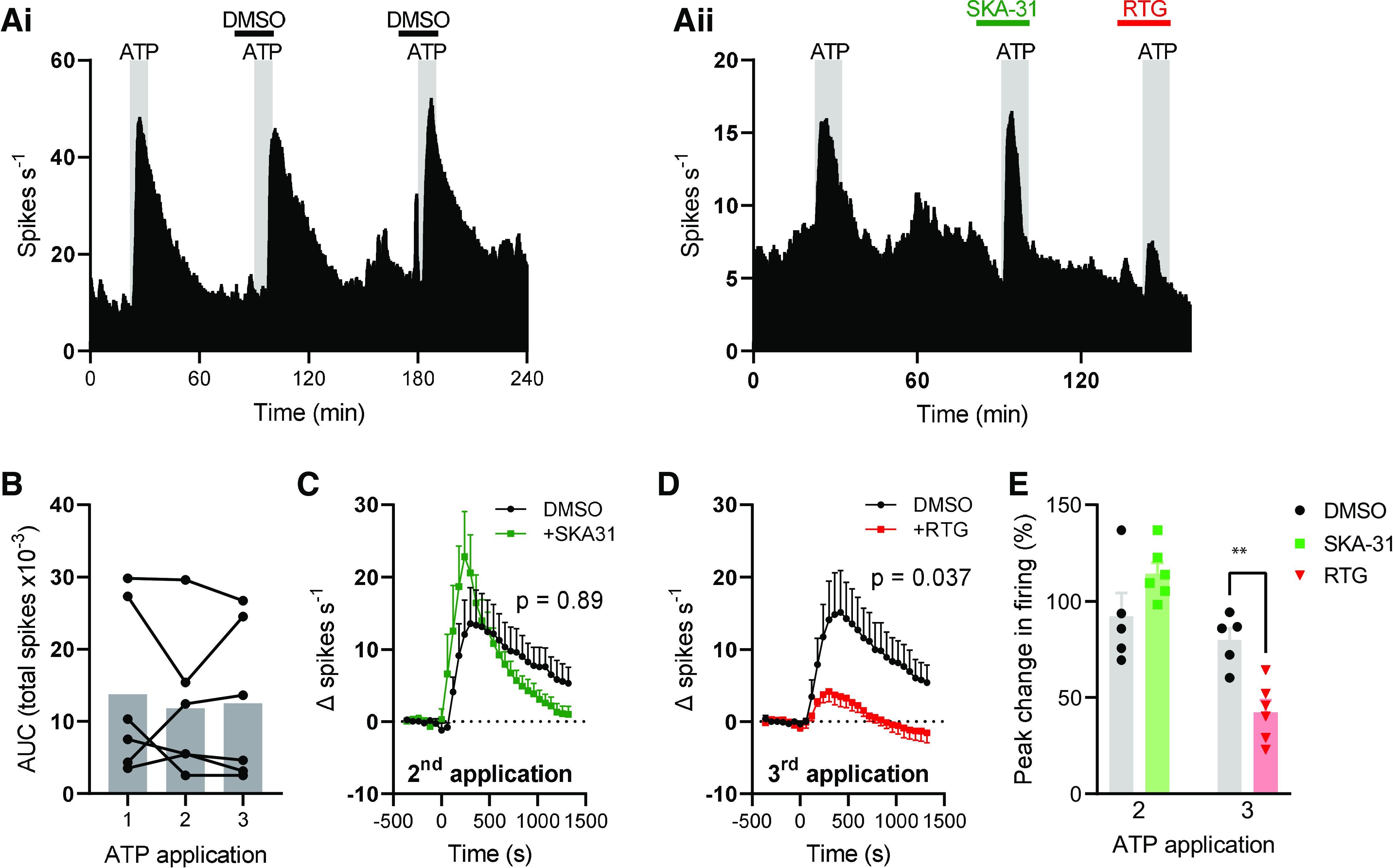
Small/intermediate conductance Ca^2+^-activated K^+^ (SK/IK) channels do not regulate the afferent response to ATP. Exemplar rate histograms showing afferent firing during repeated applications of ATP *(Ai)* with SKA-31 (*Aii*) and retigabine (RTG, *Aii*). *B*: grouped data showing the total spike discharge following ATP application for each of the three applications. Bar shows mean, filled circles show individual experiments. One-way repeated measures ANOVA. *C*: grouped data showing the change in afferent firing rate after the second ATP application (applied at 0 s) in DMSO- (black) and SKA-31 (green)-treated tissue. Two-way repeated-measures ANOVA. *D*: grouped data showing the change in afferent firing rate after the third ATP application (applied at 0 s) in DMSO- and retigabine (red)-treated tissue. Two-way repeated-measures ANOVA. *E*: grouped data showing the percentage change in afferent firing rate following the second and third applications of ATP. Two-tailed unpaired *t* test (second and third applications analyzed separately). **P < 0.01. AUC, area under afferent response curve.

Given the robust coexpression of *P2rx3* and *Bdkrb2* with transcripts encoding SK/IK channel subunits in colonic afferents, we used whole nerve suction electrode recording of the lumbar splanchnic nerve (LSN) innervating the distal colon to test whether these channels regulate the afferent response to ATP and bradykinin. Bath application of ATP (3 mM) evoked a marked increase in afferent activity that did not desensitize and was not affected by coapplication of DMSO (vehicle for later experiments, Fig. 2*Ai*). There was no difference in the peak afferent activity (main effect, *P* = 0.96) or the total spikes discharged [area under afferent response curve (AUC), main effect, *P* = 0.67, *n* = 5, Fig. 2*B*] between the first, second, and third applications of ATP.

SKA-31 (100 µM), an activator of SK (pEC_50_ = 5.5–5.7) and IK (pEC_50_ = 6.6) channels (15) had no effect on the afferent response to ATP (Fig. 2*Aii*). Afferent firing following the second ATP application was no different between DMSO- and SKA-31-treated tissues (main effect of drug, *P* = 0.89, Fig. 2*C*). ATP increased afferent firing rate by 92.3 ± 11.9% (*n* = 5) and 114.4 ± 5.6% (*n* = 6) in DMSO- and SKA-31-treated tissues, respectively (*P* = 0.12, Fig. 2*E*).

Retigabine (100 µM), an activator of K_V_7 channels, was coapplied with the third ATP application, resulting in a robust inhibition of the afferent response to ATP (Fig. 2*Aii*). Afferent firing after the third ATP application was markedly reduced by retigabine compared with DMSO-treated tissue (main effect of drug, *P* = 0.037, Fig. 2*D*). ATP increased afferent firing rate by 79.8 ± 6.1% (*n* = 5) and 42.4 ± 6.2% (*n* = 6) in DMSO- and retigabine-treated tissues, respectively (*P* = 0.0021, Fig. 2*E*).

### SK/IK Channels Do Not Regulate the Afferent Response to Bradykinin

**Figure 3.**
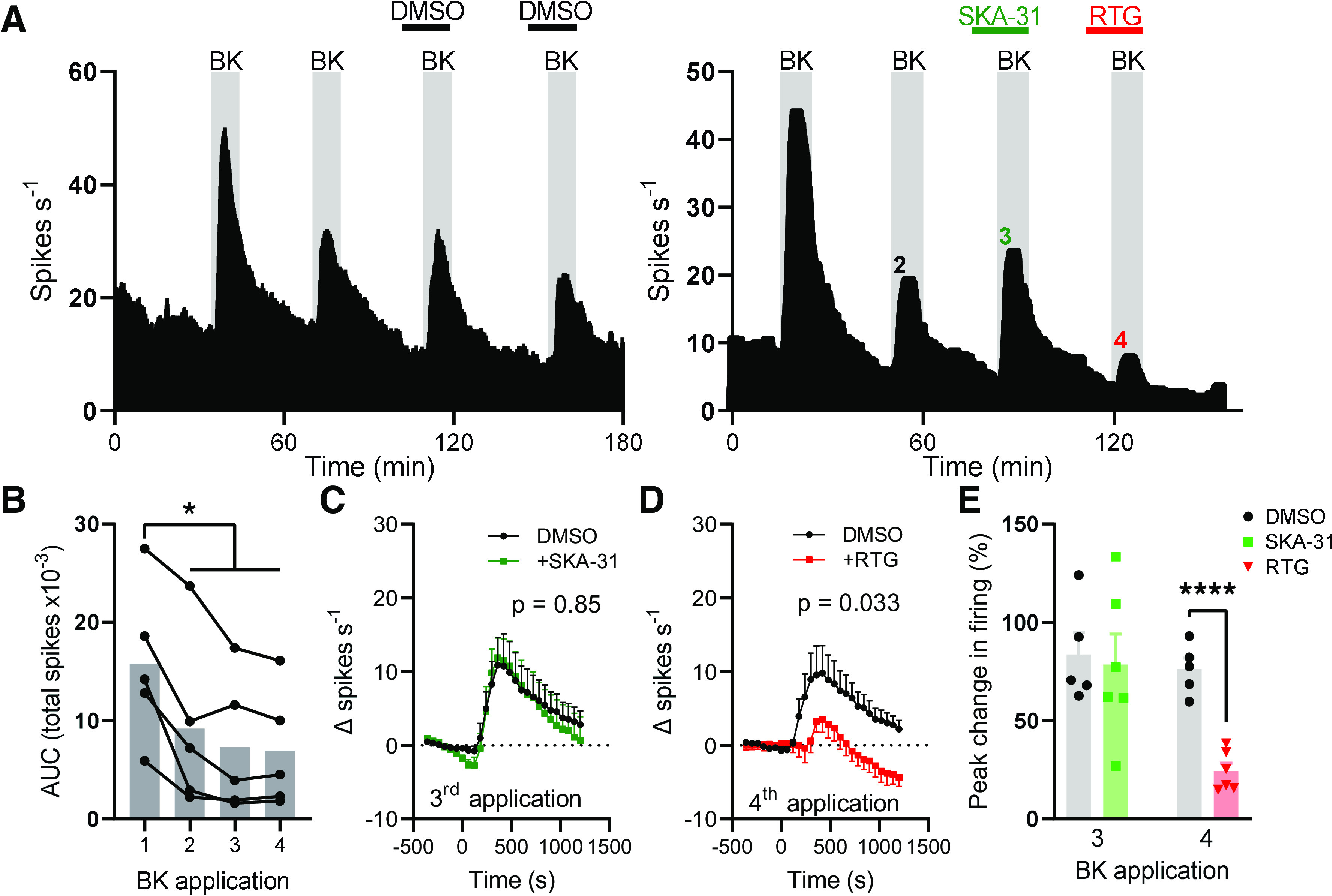
SKA-31 had no effect on bradykinin-evoked colonic afferent activity. *A*: exemplar rate histograms showing afferent firing during repeated applications of bradykinin with DMSO (BK, *left*) or with SKA-31 and retigabine (RTG, *right*). *B*: grouped data showing the total spike discharge following four successive applications of bradykinin. Bar shows mean, filled circles show individual experiments. One-way repeated-measures ANOVA with Tukey’s posttests. *C*: grouped data showing the change in afferent firing rate following the third application of bradykinin (applied at 0 s) in DMSO- and SKA-treated tissue. Two-way repeated-measures ANOVA. *D*: grouped data showing the change in afferent firing rate following the fourth application of bradykinin (applied a 0 s) in DMSO- and retigabine-treated tissue. Effect of retigabine on spontaneous activity subtracted from trace for time < 0 s. Two-way repeated-measures ANOVA. *E*: grouped data showing the percentage increase in afferent firing rate following the third and fourth application of bradykinin. Two-tailed unpaired *t* test (third and fourth applications analyzed separately). *P < 0.05, ****P < 0.0001. AUC, area under afferent response curve.

Bath application of bradykinin (1 µM) gave rise to elevated afferent activity that underwent desensitization with repeated applications (Fig. 3*A*, *left*). The first application of bradykinin evoked greater afferent firing than all subsequent applications (*P* < 0.037), but there was no difference in afferent firing evoked by the second, third, and fourth applications (*P* > 0.49, *n* = 5, Fig. 3*B*). SKA-31 (100 µM) was applied with the third application of bradykinin (Fig. 3*A*, *right*). Compared with DMSO-treated tissues, SKA-31 treatment did not affect afferent firing following bradykinin application (main effect of drug, *P* = 0.85, Fig. 3*C*).

Unlike SKA-31, retigabine pretreatment before the fourth bradykinin application inhibited the response to bradykinin (Fig. 3*A*, *right*). Bradykinin-evoked afferent firing was reduced in retigabine-treated compared with DMSO-treated tissues (main effect of drug, *P* = 0.033, Fig. 3*D*). During the third application of bradykinin, afferent firing rate increased by 83.6 ± 11.4% (*n* = 5) and 78.5 ± 15.5% (*n* = 6) in DMSO- and SKA-31-treated tissues, respectively (*P* = 0.80, Fig. 3*E*). During the fourth application of bradykinin, afferent firing increased by 76.2 ± 5.7% (*n* = 5) and 24.3 ± 4.1% (*n* = 6) in DMSO- and retigabine-treated tissues, respectively (*P* < 0.0001, Fig. 3*E*).

### SK/IK Channels Do Not Regulate the Afferent Response to Ramp Distention of the Colon

**Figure 4.**
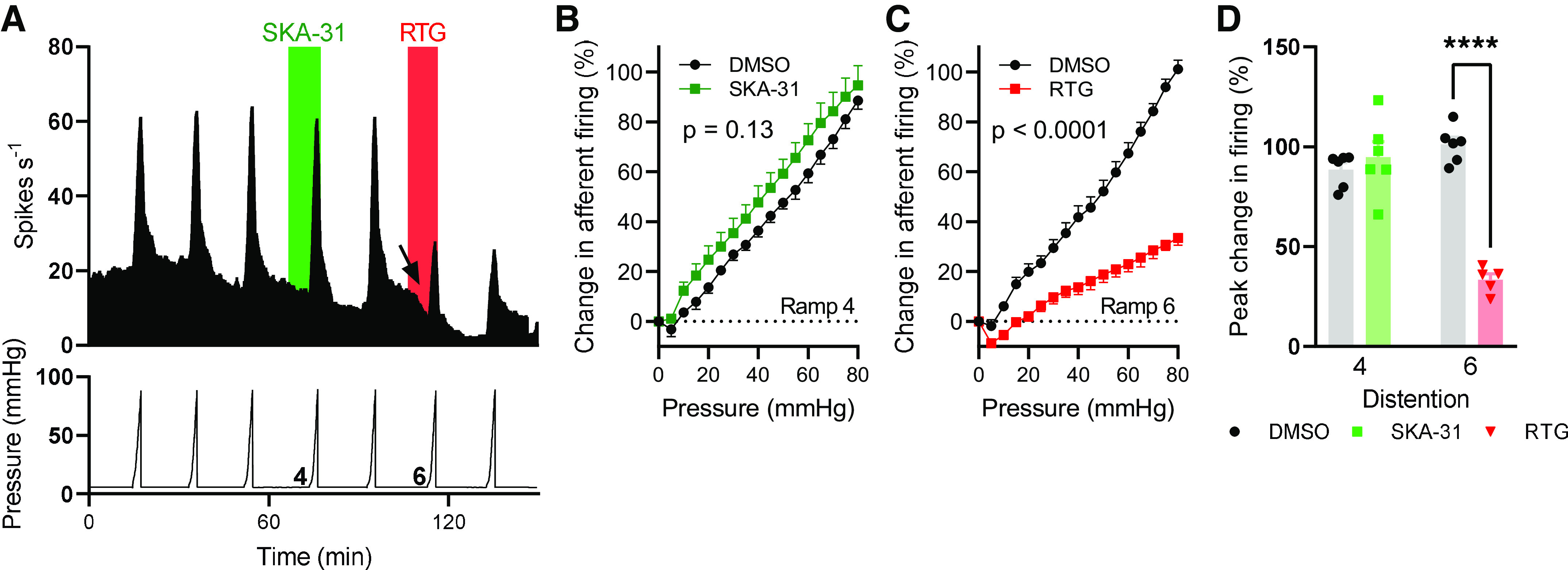
SKA-31 did not affect the afferent response to ramp distention of the colon. *A*: exemplar rate histogram (*top*) showing the afferent firing during repeated ramp distention of the colon to ∼80 mmHg luminal pressure (*bottom*). The application of SKA-31 and retigabine is shown in green and red, respectively. The arrow highlights the decrease in spontaneous afferent firing following retigabine application. *B*: pressure-response relationship for the fourth ramp distention in DMSO- and SKA-31-treated tissue. Two-way repeated-measures ANOVA. *C*: pressure-response relationship for the sixth ramp distention in DMSO- and retigabine-treated tissue. Two-way repeated-measures ANOVA. *D*: grouped data showing the percentage increase in afferent firing rate during the fourth and sixth ramp distention. ****P < 0.0001, two-tailed unpaired *t* test (fourth and sixth ramp distention analyzed separately).

Ramp distention of the colon to a luminal pressure of ∼80 mmHg resulted in repeatable increases in afferent discharge (Fig. 4*A*). Application of SKA-31 (100 µM) before and during the fourth ramp distention had no effect on pressure-induced afferent firing compared with the preceding ramp distention (Fig. 4*A*) or to DMSO-treated tissue (main effect of drug, *P* = 0.13, Fig. 4*B*). Application of retigabine before and during the sixth ramp distention reduced pressure-induced afferent firing compared with the preceding ramp distention (Fig. 4*A*) and to DMSO-treated tissue (main effect of drug, *P* < 0.0001, Fig. 4*C*). Retigabine inhibited afferent firing at luminal pressures >5 mmHg. During the fourth ramp distention, afferent activity increased 88.5 ± 3.4% (*n* = 6) and 94.7 ± 7.7% (*n* = 6) in DMSO- and SKA-31-treated preparations, respectively (*P* = 0.48, Fig. 4*D*). During the sixth ramp distention, afferent activity increased 101.0 ± 3.7% (*n* = 6) and 33.4 ± 2.9% (*n* = 5) in DMSO- and retigabine-treated preparations, respectively (*P* < 0.0001, Fig. 4*D*).

### SK/IK Channels Do Not Regulate Spontaneous Colonic Afferent Firing

**Figure 5.**
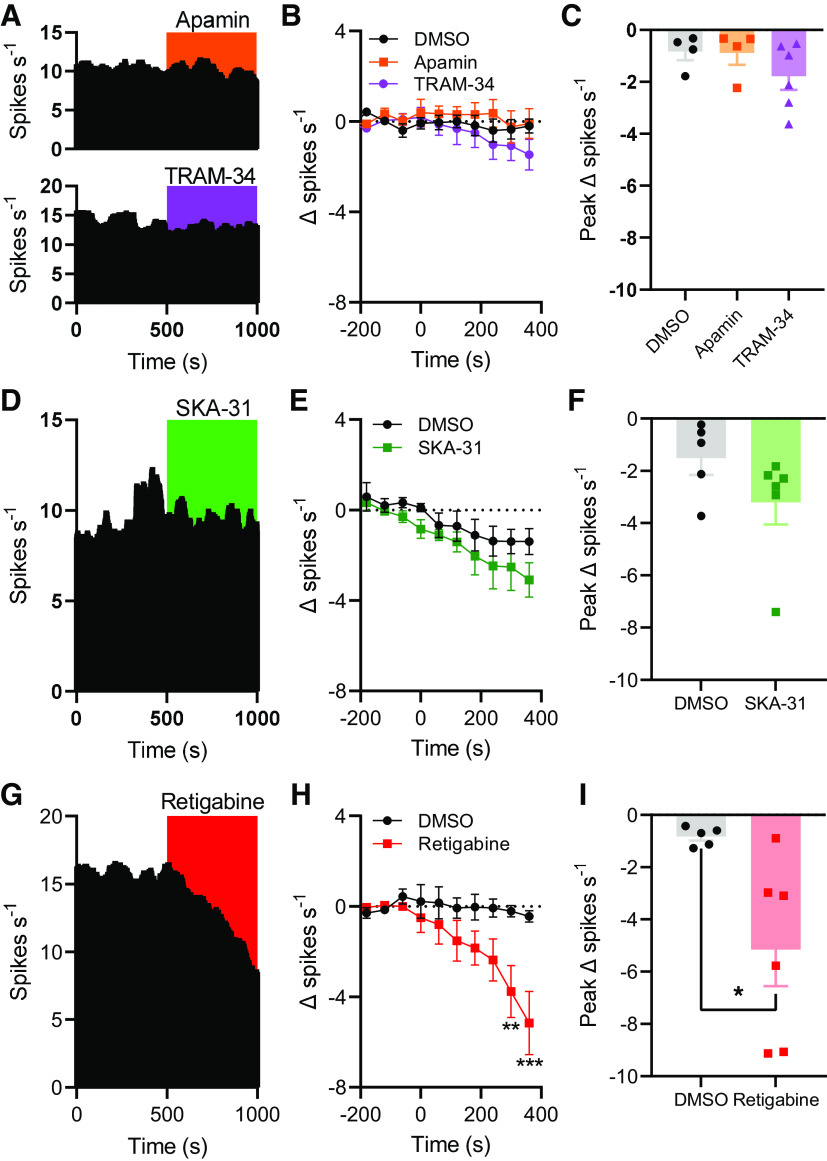
Small/intermediate conductance Ca^2+^-activated K^+^ (SK/IK) channels do not regulate spontaneous colonic afferent activity. *A*, *top*: example rate histogram showing afferent activity during the application of apamin. *A*, *bottom:* example rate histogram showing afferent activity during the application of TRAM-34. *B*: grouped data showing the change in spontaneous afferent firing rate following the application of DMSO (black), apamin (orange), or TRAM-34 (purple) (applied at 0 s). Two-way repeated-measures ANOVA. *C*: grouped data showing the peak change in afferent firing rate following DMSO, apamin, or TRAM-34 application. One-way ANOVA with Holm–Sidak’s posttests. *D*: example rate histogram showing afferent activity during the application of SKA-31. *E*: grouped data showing the change in spontaneous afferent firing rate following the application of DMSO or SKA-31 (green). Two-way repeated-measures ANOVA. *F*: grouped data showing the peak change in afferent firing rate following DMSO or SKA-31 application. Two-tailed unpaired *t* test. *G*: example rate histogram showing afferent activity during the application of retigabine. *H*: grouped data showing the change in spontaneous afferent firing rate following the application of DMSO or retigabine (red). Two-way repeated-measures ANOVA with Holm–Sidak posttests. *I*: grouped data showing the peak change in afferent firing rate following the application of DMSO or retigabine. Two-tailed unpaired *t* test. *P < 0.05, **P < 0.01, ***P < 0.001.

We next investigated whether SK/IK channels tonically regulated spontaneous colonic afferent firing by recording ongoing afferent activity before and after the application of either apamin (SK channel blocker, 1 µM, Fig. 5*A*, *top*) or TRAM-34 (IK channel blocker, 10 µM, Fig. 5*A*, *bottom*). Application of apamin failed to affect afferent activity compared with DMSO application (main effect of drug, *P* = 0.55, Fig. 5*B*). Afferent firing rate was decreased by 0.83 ± 0.33 spikes·s^−1^ (*n* = 4) and 0.89 ± 0.45 spikes·s^−1^ (*n* = 4) following the application of DMSO and apamin, respectively (*P* = 0.94, Fig. 5*C*). Similarly, TRAM-34 did not evoke afferent discharge (main effect of drug, *P* = 0.55, Fig. 5*B*). The peak change in afferent firing following TRAM-34 application (−1.8 ± 0.52 spikes·s^−1^, *n* = 6) was no different from that in vehicle control experiments (*P* = 0.45, Fig. 5*C*).

Consistent with these findings, and those from earlier experiments, SKA-31 application (Fig. 5*D*) had no effect on spontaneous nerve activity compared with DMSO-treated tissue (main effect of drug, *P* = 0.22, Fig. 5*E*). The peak decrease in afferent firing rate after SKA-31 application (3.2 ± 0.7 spikes·s^−1^, *n* = 6) was no different from that after DMSO application (1.5 ± 0.6 spikes·s^−1^, *n* = 5, *P* = 0.16, Fig. 5*F*).

Conversely, the application of retigabine (Fig. 5*G*) suppressed spontaneous afferent firing ≥5 min after application (main effect of drug, *P* = 0.069; 5 min, *P* = 0.0052; 6 min, *P* < 0.0001, Fig. 5*H*). The peak decrease in spontaneous afferent discharge in control experiments was 0.83 ± 0.16 spikes·s^−1^ (*n* = 5) compared with 5.2 ± 1.4 spikes·s^−1^ (*n* = 6) after retigabine application (*P* = 0.027, Fig. 5*I*).

### SK/IK Channels Regulate Colonic Motility

**Figure 6.**
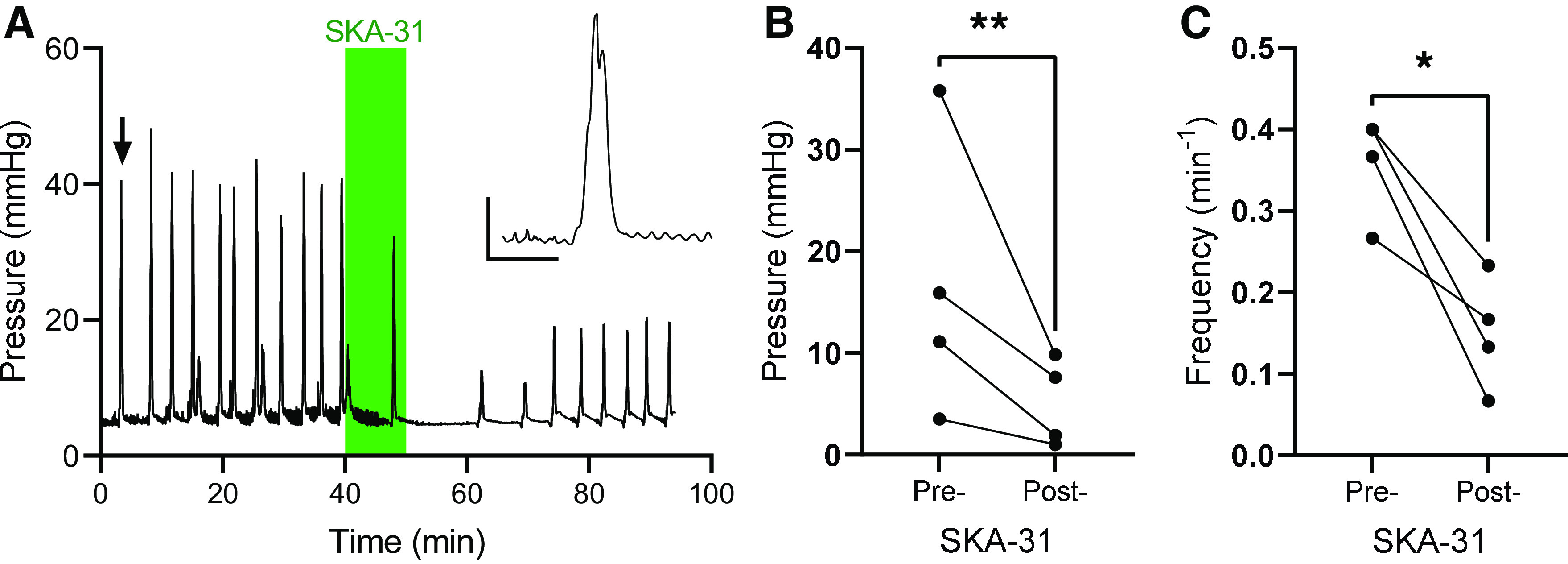
Small/intermediate conductance Ca^2+^-activated K^+^ (SK/IK) channels regulate colonic motility. *A*: exemplar recording of colonic luminal pressure, showing the effect of SKA-31 application (green shaded area). *Inset*: expanded trace showing an individual colonic migrating motor complex (CMMC) (highlighted by the arrow). Scale: 1 min; 10 mmHg. *B*: grouped data showing the amplitude of CMMCs before and after the application of SKA-31. **P < 0.01, two-tailed ratio-paired *t* test. *C*: grouped data showing the frequency of CMMCs before and after the application of SKA-31. *P < 0.05, two-tailed ratio-paired *t* test.

Finally, to confirm the activity of SKA-31, we demonstrated its ability to inhibit ongoing peristaltic gut motility consistent with the known role of IK channels in mediating the afterhyperpolarization of Dogiel type II enteric neurons. Pressure changes evoked by colonic migrating motor complexes (CMMCs) were recorded before and after the application of SKA-31 (100 µM, Fig. 6*A*). CMMCs exhibited an average amplitude 16.6 ± 6.9 mmHg before SKA-31 application and 5.1 ± 2.2 mmHg after SKA-31 application (*P* = 0.0092, *n* = 4, Fig. 6*B*). The frequency of CMMCs was also reduced from 0.35 ± 0.03 min^−1^ to 0.15 ± 0.03 min^−1^ by SKA-31 application (*P* = 0.045, *n* = 4, Fig. 6*C*).

## DISCUSSION

We have investigated the role of SK and IK channels in regulating the colonic afferent response to ATP, bradykinin, and distention of the colon. ATP and bradykinin are important algogenic stimuli known to contribute to visceral nociception and pain during inflammation. Distention of the colon is an important visceral stimulus in both physiological and pathological states. The colonic afferent response to all of these stimuli, as well as basal afferent activity, was unaffected by the opening of SK/IK channels by SKA-31.

This observation was unexpected given the expression of transcripts encoding SK channel subunits in back-labeled colonic afferent neuron cell bodies (13). However, the expression data used here does not give any indication of protein expression at the afferent terminals in the colon. It may be that the cell bodies and terminals of colonic afferents express different subsets of ion channels and that SK channels *are* only expressed at the cell body. If SK channels *are* expressed at the terminals of colonic afferent nerves, why did SKA-31 have no effect on afferent activity? It is possible that SK channels are tonically active under basal conditions (16, 17), rendering SKA-31 unable to potentiate their activity any further. However, inhibition of SK channels with apamin did not enhance afferent activity, suggesting that SK channels are unlikely to be tonically active. The inhibition of IK channels with TRAM-34 also yielded no change in colonic afferent activity, indicating that IK channels are not likely tonically active either.

It is also possible for SK channel subunits form heteromeric channels with other members of the SK family, which can affect trafficking of channels to the membrane (5) and channel pharmacology (4, 5, 18). For example, channels formed of SK1 and SK2 subunits exhibited an apamin sensitivity intermediate between SK1 and SK2 (4, 18). The high concentration of apamin used in the present study would still be expected to robustly inhibit heteromeric channels. Therefore, it is doubtful that the lack of effect of apamin observed here is result of the formation of heteromeric SK channels. SK1 subunits preferentially coassemble with IK (rather than forming homomeric channels), forming SK1-IK channels insensitive to apamin and with a markedly reduced sensitivity to TRAM-34 compared with homomeric IK channels (19). Approximately 80% of *Kcnn4*-expressing colonic afferents in mouse coexpress *Kcnn1*, indicating the potential for the presence of a significant population of heteromeric SK1-IK channels. Despite this, if functional, tonically active SK/IK channels were expressed at the afferent terminals in the manner suggested by expression at the cell bodies (13), it would still be expected that colonic afferent activity could be elevated by apamin or TRAM-34. This is because *1*) a large population of *Kcnn1*-expressing afferents do not express *Kcnn4*, and *2*) 10 µM TRAM-34 (used in this study) blocks ∼50% of heteromeric SK1-IK channel current (19), which would be expected to induce membrane depolarization sufficient to trigger action potential discharge. Consequently, it seems unlikely that heteromeric channel formation underpins the lack of effect of apamin and TRAM-34 on colonic afferent activity. The effect of SKA-31 on heteromeric SK/IK channels is not known.

In all, it seems probable that the lack of effect of SKA-31, apamin, and TRAM-34 is due to a lack of SK and IK channel expression on colonic afferent terminals. SK channels expressed in sensory neuron cell bodies could still control visceral sensory input to the central nervous system (9, 20), but they do not appear to contribute to the control of colonic afferent activity at the terminals.

At odds with results obtained with SKA-31, we found that retigabine inhibited colonic afferent activity in response to stimulation with ATP, bradykinin, and distention of the colon. Transcripts encoding receptors for ATP and bradykinin are frequently coexpressed with transcripts encoding K_V_7 channels subunits (*KCNQ2*, *KCNQ3*, and *KCNQ5*) (12, 13). Both ATP and bradykinin evoke action potential firing through multiple mechanisms, including the inhibition of K_V_7 channels in a phospholipase C- (PLC) dependent manner (21, 22). It is likely that many PLC-coupled receptors, such as protease-activated receptor 2 (PAR2) (23) and Mas-related G protein-coupled receptor member D (MrgD) (24), stimulate action potential discharge through the inhibition of K_V_7 channels. Suppression of K_V_7 channel activity triggers membrane depolarization necessary for the activation of voltage-gated Na^+^ (Na_V_) channels required to further amplify depolarizations (e.g., Na_V_1.9) and trigger action potential generation (e.g., Na_V_1.8). Indeed, nocifensive behaviors evoked by bradykinin injection and colonic afferent discharge evoked by ATP can both be markedly attenuated by genetic loss of Na_V_1.9 (14, 21). It seems clear that K_V_7 channels are an important regulator of the colonic afferent response to chemical stimuli.

Retigabine also robustly inhibited the afferent response to distention of the colon across a wide range of distention pressures (>5 mmHg). This indicates that K_V_7 channels likely regulate the function of low-threshold, wide dynamic range, and high-threshold mechanosensitive afferents, in agreement with the broad expression of transcripts encoding the subunits comprising K_V_7 channels (12, 13). It has previously been shown that retigabine suppressed mechanically evoked activity in the saphenous nerve (25) and that linopiridine, a K_V_7 channel blocker, potentiated the activity of rapidly adapting mechanoreceptors in the skin (26).

Finally, we observed a robust suppression of the amplitude and frequency of CMMCs following the application of SKA-31, consistent with the well-established role for IK channels in the regulation of the excitability of Dogiel type II neurons (27) that initiate peristaltic reflexes. Opening of SK or IK channels causes membrane hyperpolarization, suppressing voltage-gated Ca^2+^ channel activity and, hence, reduces the Ca^2+^ influx required for smooth muscle contraction. SK channel expression has already been identified in colonic smooth muscle (28). The tension of colonic muscle was increased by SK channel blockade, and estrogen-induced colonic smooth muscle relaxation may be due, in part, to the opening of SK channels (29).

In summary, data presented here do not support the hypothesis that SK/IK channels regulate colonic afferent activity, though these channels do appear to regulate colonic smooth muscle activity. Our data do, however, provide further support for K_V_7 channels playing a key role in regulating the colonic afferent response to chemical and mechanical stimuli.

## DATA AVAILABILITY

Source data for this paper are freely available through Figshare at https://doi.org/10.6084/m9.figshare.23643621.v1.

## GRANTS

This work was supported by a Crohn’s and Colitis United Kingdom Grant PC2019/1-Bulmer (to D.C. Bulmer). C.N. Bhebhe was supported by a Gates Cambridge Trust scholarship.

## DISCLOSURES

No conflicts of interest, financial or otherwise, are declared by the authors.

## AUTHOR CONTRIBUTIONS

C.N.B., T.R., and D.C.B. conceived and designed research; C.N.B. and R.A.G. performed experiments; C.N.B. and J.P.H. analyzed data; C.N.B., J.P.H., and D.C.B. interpreted results of experiments; C.N.B., J.P.H., and D.C.B. prepared figures; J.P.H. and D.C.B. drafted manuscript; C.N.B., J.P.H., R.A.G., T.R., and D.C.B. edited and revised manuscript; C.N.B., J.P.H., R.A.G., T.R., and D.C.B. approved final version of manuscript.
